# Evaluation of an e-learning on involving significant others in occupational health care

**DOI:** 10.1093/eurpub/ckac129.313

**Published:** 2022-10-25

**Authors:** NC Snippen, HJ de Vries, M Hagedoorn, S Brouwer

**Affiliations:** Department of Health Sciences, University Medical Center Groningen, Groningen, Netherlands

## Abstract

**Background:**

Significant others (SOs) like partners, family members or friends can play an important role in how workers cope with chronic disease, thereby influencing work and health outcomes. Despite the potential benefits of involving SOs in the return-to-work (RTW) process of workers with chronic diseases, guidance and training on how to manage this is scarce. Educating work and health professionals on this topic could lead to better RTW support for workers and prevention of long-term sickness absence.

**Objectives:**

This study aimed to determine the effectiveness of an e-learning for improving occupational health physicians’ (OHPs) knowledge, attitudes, and self-efficacy regarding involving SOs in the RTW process. In addition, we explored OHPs’ responses to and satisfaction with the e-learning. We conducted a randomized controlled trial with 87 OHPs, involving an intervention group and a waitlisted control group. Between-group differences in knowledge, attitude, self-efficacy outcomes, and retention of effects were assessed using ANOVA and paired t-tests. Reactions to the e-learning were analyzed with descriptive statistics and thematic analysis.

**Results:**

We found moderate to large effects on OHPs’ knowledge (p <.001, ηp2 = .202), attitudes (ηp2 = .098), and self-efficacy (p < .001, ηp2 = .237), with retention of all changes at 10-week follow-up. OHPs graded the e-learning with a mean score of 7.9 out of 10 (SD = 1.11) and indicated that the e-learning increased their awareness of the role of SOs in RTW and encouraged them to address this more often.

**Conclusions:**

The developed e-learning is the first evidence-based training to improve the knowledge, attitudes, and self-efficacy of OHPs with regard to involving SOs in the RTW process of workers with chronic diseases. The e-learning and accompanying materials can encourage work and health professionals to more often address the role of significant others in the work re-integration process.

**Key messages:**

• The developed e-learning is effective in increasing occupational health physicians’ knowledge, attitudes and self-efficacy with regard to involving significant others in the return-to-work process.

• The e-learning and accompanying materials can encourage professionals to more often address the role of significant others in the work re-integration process to prevent long-term sickness absence.

